# Identification of immune suppressor candidates utilizing comparative transcriptional profiling in histiocytic sarcoma

**DOI:** 10.1007/s00262-024-03908-x

**Published:** 2025-01-03

**Authors:** Jennifer A. Lenz, Brandon Peng, Charles‑Antoine Assenmacher, Austin King, Paul J. Zhang, Robert G. Maki, M. Andres Blanco, Enrico Radaelli, Matthew J. Atherton

**Affiliations:** 1 Department of Clinical Sciences and Advanced Medicine, School of Veterinary Medicine, University of Pennsylvania, Philadelphia, PA USA; 2 Department of Biomedical Sciences, School of Veterinary Medicine, University of Pennsylvania, Philadelphia, PA USA; 3 Department of Pathobiology, School of Veterinary Medicine, University of Pennsylvania, Philadelphia, PA USA; 4 Department of Pathology and Laboratory Medicine, Perelman School of Medicine, University of Pennsylvania, Philadelphia, PA USA; 5 Memorial Sloan Kettering Cancer Center, New York, NY USA

**Keywords:** Histiocytic sarcoma, Osteopontin, PD-1, TXNIP

## Abstract

Histiocytic sarcoma (HS) is a rare yet lethal malignancy with no established standard of care therapies. A lack of pre-clinical models limits our understanding of HS pathogenesis and identification of therapeutic targets. Canine HS shares multiple clinical and genetic similarities with human HS, supporting its use as a unique translational model. Prior studies have investigated the immunogenicity of HS. Although increased tumor infiltrating lymphocyte (TIL) density is associated with favorable outcomes in canine HS, virtually all canine patients eventually succumb to progressive disease consistent with ultimate failure of anti-tumor immunity. To investigate potential regulators of the immune tumor microenvironment (TME), we undertook a comparative transcriptional approach of three long-lived cases of canine pulmonary HS with heavy T cell infiltrate and three short-lived cases of splenic HS that lacked significant T cell inflammation and compared these data to corresponding grossly normal tissues from dogs undergoing necropsy. This comparison identified *PDCD1,* encoding the immune checkpoint PD-1, and *SPP1,* encoding the secreted pro-tumorigenic protein osteopontin, as positive differentially expressed genes (DEGs) in canine HS. *TXNIP,* encoding the tumor suppressor TXNIP, was the most significant negative DEG. Comparative transcriptomic studies revealed conservation of enriched (including *SPP1*) and depleted (including *TXNIP*) DEGs between canine and human HS patients. Immunohistochemistry demonstrated osteopontin in the TMEs of canine and human HS. Collectively, we uncover PD-1, osteopontin, and TXNIP as putative actionable targets in HS and further establish canine HS as a preclinical platform to screen novel immunotherapeutic approaches for this deadly disease.

**Supplementary Information:**

The online version contains supplementary material available at 10.1007/s00262-024-03908-x.

## Introduction

Histiocytic sarcoma (HS) is a remarkably rare but highly aggressive tumor of non-Langerhans histiocytes, with a reported median survival time of 6 months following diagnosis [1]. Unfortunately, the paucity of widely available preclinical HS models has hindered identification of therapeutic targets that could improve the prognosis of HS patients. Canine HS shares multiple clinical and genetic similarities with human HS, and as specific breeds are predisposed to HS, it is frequently observed in veterinary medicine [2, 3]. Such findings identify pet dogs as a unique setting in which to identify and assess potential therapeutics for spontaneous HS.

Our previous work highlighted the immunogenicity of canine HS, and its overlapping spectrum of tumor infiltrating lymphocyte (TIL) density with that observed in human HS supports preserved cross species biology [4]. Although increased TIL density was associated with better outcomes in canine HS, virtually all dogs eventually succumb to their disease, suggesting the ultimate failure of anti-tumor immunity. To investigate potential regulators of immunity in the tumor microenvironment (TME), we performed transcriptional profiling on exceptional and poor survivors from our previously published cohort and compared these data to corresponding grossly normal tissues from dogs without cancer undergoing necropsy [4]. We then documented conservation of differentially expressed genes (DEGs) between species and found that *SPP1*, encoding for osteopontin, was enriched in both canine and human HS. Expression of osteopontin was confirmed in canine and human HS using immunohistochemistry (IHC). As osteopontin has T cell suppressive activity in other malignancies, our data implicate osteopontin as a potential regulator of anti-tumor immunity in canine and human HS, and identifies multiple other candidate genes with immunomodulatory potential [5, 6].

## Materials and methods

### Study populations

All canine and human HS samples were previously reviewed by board certified pathologists confirming the diagnosis with appropriate immunohistochemical markers [4]. Transcriptional profiling was performed on tumors from three dogs with canine pulmonary HS with heavy T cell infiltrate and prolonged survival times, and three other dogs with splenic HS that lacked significant T cell inflammation and had short survival times as previously described [4]. All grossly normal tissues were trimmed from HS tissues prior to RNA extraction. Transcriptional analyses of RNA from grossly normal lung and splenic tissues collected from three unrelated dogs without neoplasia undergoing routine post-mortem examination was also undertaken (Table S1). Immunohistochemistry was performed on all biopsies of our previously described cohort of 18 canine HS and 5 human HS patients [4]. These studies were exempt from review for the University of Pennsylvania’s Institutional Animal Care and Use Committee and the University of Pennsylvania Veterinary Schools Privately Owned Animal Protocol Committee. Analysis of human tissues was approved by the University of Pennsylvania’s Institutional Review Board (IRB#848935).

### NanoString transcriptional profiling

RNA was extracted from scrolls cut from formalin-fixed paraffin-embedded (FFPE) tissues using RNeasy FFPE kits (Qiagen) prior to quantification using Qubit (Invitrogen) and TapeStation (Agilent) as previously described [4]. 100ngs of total RNA was hybridized using the nCounter Canine IO Panel and data were acquired using the nCounter Flex system [4]. Data was analyzed by ROSALIND® (https://rosalind.bio/), with a HyperScale architecture developed by ROSALIND, Inc. (San Diego, CA). Normalization, fold changes, and p values for DEGs were calculated using criteria provided by Nanostring. ROSALIND® follows the nCounter® Advanced Analysis protocol of dividing counts within a lane by the geometric mean of the normalizer probes from the same lane. Housekeeping probes to be used for normalization were selected based on the geNorm algorithm as implemented in the NormqPCR R library [7]. p value adjustment was performed using the Benjamini–Hochberg method of estimating false discovery rates (FDR) when comparing all HS samples to all normal samples. Clustering of genes for the final heatmap of differentially expressed genes was done using the PAM (Partitioning Around Medoids) method using the fpc R library (https://cran.r-project.org/web/packages/fpc/index.html) that takes into consideration the direction and type of all signals on a pathway, the position, role, and type of every gene, etc. Differentially expressed genes were reported when fold change was ≥ 1.5 or ≤ −1.5 and were considered statistically significant when p-Adj ≤ 0.05 when comparing all six HS vs. all six control tissues, or when p ≤ 0.01 when comparing three HS from distinct anatomic locations (lung or spleen) to three corresponding control tissues. Shared canine and human DEGs were identified by searching statistically significant DEGs reported by Egan et al*.* [8] in 17 cases of human HS compared with four cases of human reactive nodal histiocytic infiltrates as controls (absolute log fold change > 1, p-Adj ≤ 0.05) to the canine DEGs identified within our dataset when comparing all six canine HS to all six canine control tissues.

### Immunohistochemistry

5 μm FFPE sections were mounted and stained using Leica BOIND RXm automated platform as previously described [4]. Osteopontin was detected in both canine and human sections using primary polyclonal rabbit anti-human osteopontin antibody (Rockland Immunochemicals). Slides were scanned using an Aperio AT2 automated slide scanner (Leica Biosystems). Osteopontin expression was quantified using a positive pixel algorithm applied to outlined tumors with exclusion of necrotic areas (ImageScope software, Leica Biosystems).

### Statistical analysis

Prism 10 (GraphPad Software) was used to perform Spearmen correlations with Spearman’s rho (r_s_) and two-tailed p values reported.

## Results

### Comparative transcriptional profiling reveals multiple HS immune suppressor candidates

**Fig. 1:**
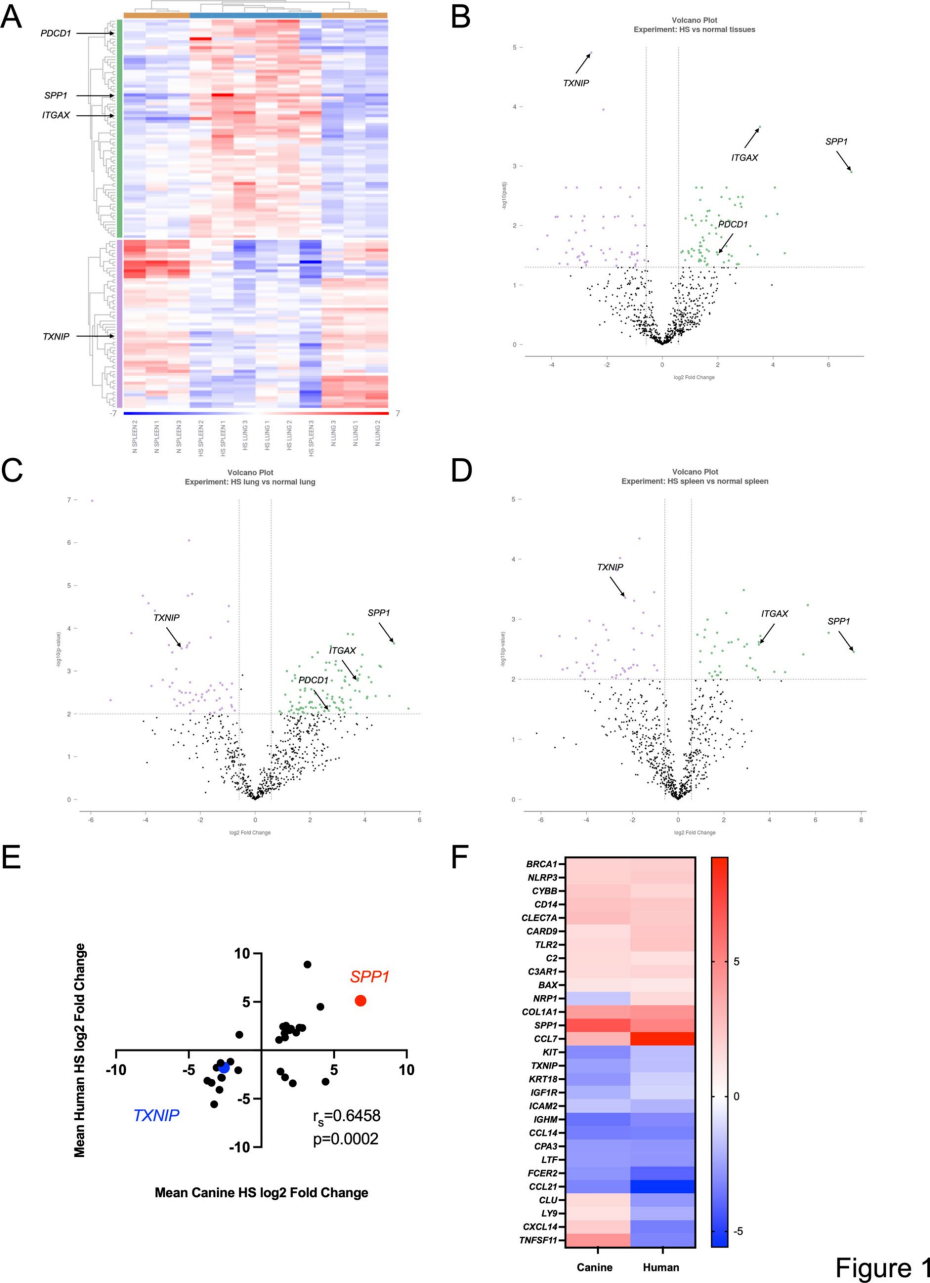
Comparative transcriptional immune analyses of histiocytic sarcoma (HS) and control tissues. Immune profiling was performed on canine tissues using the NanoString nCounter Canine IO Panel and data were analyzed using ROSALIND® platform. **A** Heatmap of genes differentially expressed (p-Adj ≤ 0.05, fold change ≥ 1.5 or ≤ − 1.5) in three normal canine spleens (N SPLEEN), three canine splenic HS tumors (HS SPLEEN), three canine pulmonary HS tumors (HS LUNG) and three normal canine lungs (N LUNG). Each column represents an individual sample, and each row represents an individual gene with shading indicating normalized expression. Volcano plots of all genes differentially expressed between **B** six canine HS tumors and six normal tissues (p-Adj ≤ 0.05, fold change ≥ 1.5 or ≤ − 1.5), **C** between three canine pulmonary HS tumors and three normal lungs (p ≤ 0.01, fold change ≥ 1.5 or ≤ − 1.5) and, **D** three canine splenic HS tumors and three normal spleens (p ≤ 0.01, fold change ≥ 1.5 or ≤ − 1.5). Each dot represents an individual gene. **E** Fold changes and Spearmen correlation of statistically significant (p-Adj ≤ 0.05) conserved DEGs across canine and human HS and **F** heatmap of the 29 significant DEGs in canine and human HS. Spearmen correlation performed using Prism 10 (GraphPad Software) with Spearman’s rho (r_s_) and two-tailed p value reported

Comparison of all tumors to all normal tissues revealed 131 DEGs (Fig. 1A, B, Table 1, Table S2). Pulmonary and splenic HS tumors exhibited transcriptional overlap and clustered together, whereas expression profiles of normal tissues differed significantly from HS tumors and exhibited differences between anatomic locations (Fig. 1A). A comparison of pulmonary HS to normal lung and splenic HS to normal spleen identified 165 and 86 DEGs respectively (Fig. 1C, D, Tables S3 and S4).

**Table 1:** Top 20 differentially expressed genes between all canine HS and all control tissues

Name	Description	Fold change	Log fold change	*p*-value	p-adj
TXNIP	Thioredoxin interacting protein	− 5.93616	− 2.56953	1.60E-08	1.23E-05
BCR	Breakpoint cluster region	− 4.38109	− 2.13129	2.91E-07	0.000112
ITGAX	Integrin, alpha X (complement component 3 receptor 4 subunit)	11.4743	3.52033	8.50E-07	0.000218
SPP1	Secreted phosphoprotein 1	113.461	6.82605	6.54E-06	0.001257
BAX	BCL2-associated X protein	2.33275	1.22203	2.61E-05	0.002292
COL1A1	Collagen, type I, alpha 1	16.7058	4.06228	2.84E-05	0.002292
CD80	CD80 molecule	2.68474	1.42478	3.58E-05	0.002292
KIT	V-kit Hardy-Zuckerman 4 feline sarcoma viral oncogene homolog	− 8.49998	− 3.08746	2.50E-05	0.002292
NRP1	Neuropilin 1	− 2.86594	− 1.51901	2.61E-05	0.002292
CREBBP	CREB binding protein	− 1.80419	− 0.85135	3.87E-05	0.002292
ITGA1	Integrin, alpha 1	− 4.67087	− 2.22369	3.76E-05	0.002292
SHMT2	Serine hydroxymethyltransferase 2 (mitochondrial)	4.97984	2.3161	1.77E-05	0.002292
PECAM1	Platelet/endothelial cell adhesion molecule 1	− 11.1743	− 3.48211	3.88E-05	0.002292
MAPK8	Mitogen-activated protein kinase 8	− 2.25018	− 1.17004	7.16E-05	0.003301
CXCR3	Chemokine (C-X-C motif) receptor 3	7.46752	2.90063	6.41E-05	0.003301
CDKN2A	Cyclin-dependent kinase inhibitor 2A (melanoma, p16, inhibits CDK4)	6.66392	2.73637	6.81E-05	0.003301
CEBPA	CCAAT/enhancer binding protein (C/EBP), alpha	4.38778	2.13349	7.30E-05	0.003301
LOC484306	Leukocyte immunoglobulin-like receptor, subfamily B (with TM and ITIM domains), member 5-like	5.37699	2.4268	8.31E-05	0.003551
TOLLIP	Toll interacting protein	2.57614	1.36521	9.15E-05	0.003705
CLEC7A	C-type lectin domain family 7, member A	7.05952	2.81957	0.000111	0.004266

Profiling revealed that *ITGAX* encoding for CD11c exhibited the most significant increase in expression when comparing all HS tumors to all normal tissues, as well as in the separate pulmonary and splenic analyses (Figs. 1,S1, S2, Table 1, Tables S2–S4). This result was not surprising, as CD11c is expressed by canine interstitial dendritic cells (DCs) which represent the cell of origin in canine HS, and CD11c expression is similarly observed in human HS [3, 9, 10].

In light of the immunogenic potential of HS, we sought to identify transcripts that could represent actionable targets [4]. *PDCD1* encoding for the immune checkpoint protein PD-1 exhibited positive differential expression in all tumors compared to all normal tissue, and in the sub-analysis of long-lived T cell inflamed pulmonary HS compared to healthy lung. However, there was no significant difference when short-lived sparsely T cell inflamed splenic HS was compared to normal spleens, suggesting that PD-1 was preferentially upregulated in tumors exhibiting increased TIL density. Accordingly, normalized *PDCD1* expression was positively associated with canine HS survival times (Figs. 1, S1–S3, Tables S2–S4). Further analysis identified *TXNIP*, the gene encoding thioredoxin-interacting protein (TXNIP), as the most significant negative DEG in all HS compared with all controls alongside significantly decreased expression in both splenic and pulmonary sub-analyses (Figs. 1A-D,S1,  S2, Table 1, Tables S2–S4). TXNIP plays a major role in regulating cellular reduction–oxidation reactions and acts as a tumor suppressor in multiple malignancies [11]. Finally, *SPP1* (encoding for osteopontin) had the greatest positive fold change observed in all HS tumors compared with all controls (Fig. 1A, B, Table 1, Table S2). *SPP1* remained significantly elevated in subset analyses of both locations (Fig. 1C, D, Tables S3, S4). As osteopontin is a multi-functional, pro-tumorigenic, secreted sialoprotein that is produced by various cells including osteocytes, tumor cells, and multiple hematopoietic cells including DCs, it could potentially contribute to the aggressive behavior of HS [12].

We next sought to identify conserved DEGs between canine and human HS and we documented 29 significant DEGs between our data when comparing all canine HS to all canine control tissues to the transcriptomic data reported by Egan et al. [8]*.* Of the 29 DEGs, 83% were either commonly enriched (including *SPP1*) or depleted (including *TXNIP*) when comparing between neoplastic and control tissues with a significant and strong correlation detected between the two datasets (Fig. 1E, F, Table S5). Collectively our data revealed conserved transcriptional changes in the immune TME in canine and human HS.

### Immunohistochemistry confirms osteopontin expression in canine and human HS

**Fig. 2:**
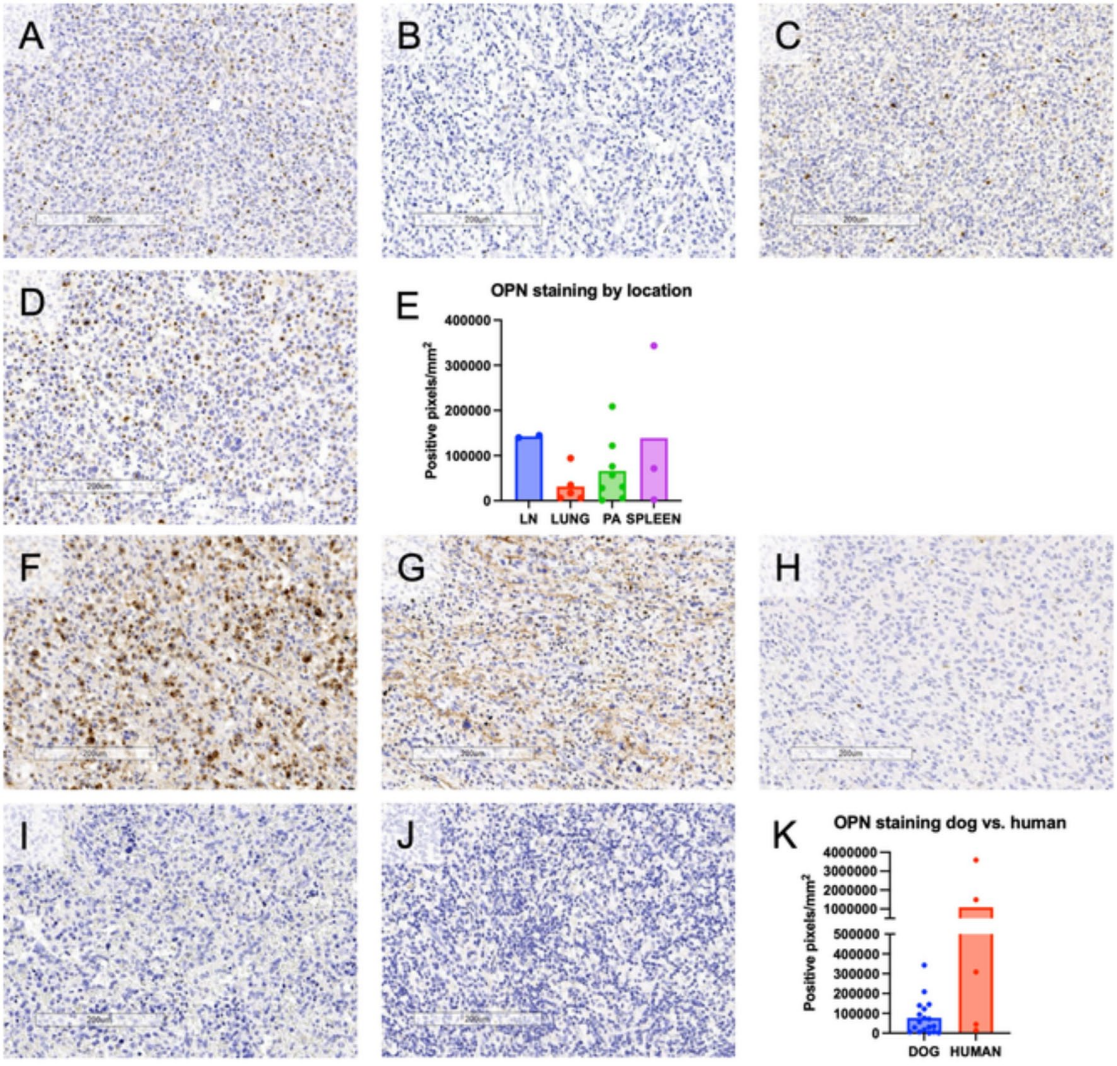
Immunostaining of canine and human histiocytic sarcoma (HS) for osteopontin. Formalin-fixed paraffin-embedded tissues from 18 canine HS and five human HS were stained for osteopontin expression. Slides scanned using an Aperio AT2 automated slide scanner (Leica Biosystems) were visualized using ImageScope software (Leica Biosystems) and an algorithm for quantifying positive pixels was applied to non-necrotic areas of the entirety of the outlined tumor area. Photomicrographs of representative canine HS with primary tissue of origin of **A** lymph node (LN), **B** lung, **C** peri-articular (PA) tissues and **D** spleen. **E** Quantification of osteopontin staining grouped by anatomic location in 18 canine HS samples. Each dot represents an individual tumor, and the bar represents the anatomic mean. Photomicrographs of human tumors from five patients diagnosed with HS in **F** the brain, **G** subcutaneous tissue of the buttock, **H** subcutaneous tissue of the foot, **I** spleen and **J** tonsil. **K** Quantification of osteopontin staining grouped by species. Each dot represents an individual tumor, and the bar represents the species mean. Scale bars measure 200 μm

To determine if protein levels of osteopontin reflected our transcriptomic findings, we performed immunohistochemistry using tumor samples from our previously published cohort. Sections from 18 localized canine HS tumors from varied anatomic locations were stained and quantified for osteopontin expression using an antibody originally raised against human osteopontin with cross reactivity for canine osteopontin (Fig. S4). A range of positivity was documented across anatomic locations in canine HS (Fig. 2A–E). We utilized the same IHC protocols and reagents to stain five human HS samples described previously to assess the translational relevance of our findings for human HS [4]. While a range of staining intensities was also observed in human HS, we found particularly high expression in two HS patients with lesions in the brain and a subcutaneous site (Fig. 2F–K).

## Discussion

The rarity of human patients diagnosed with HS effectively precludes the performance of meaningful prospective trials to determine standard-of-care treatments. As such, there is a pressing need to develop accurate model systems to discover and appraise novel therapeutic targets for this disease. Amongst the genes that we report, *ITGAX* encoding CD11c was the most significant positive DEG between HS and normal tissues, reflecting the HS cell of origin. We also identified *SPP1* as the DEG expressing the greatest positive fold change and confirmed the expression of osteopontin at a protein level in a larger cohort of canine tumors alongside human HS using a single cross-reactive antibody. As we found a strong correlation between canine and human DEGs, collectively these data support the use of pet dogs in a comparative approach to model HS.

A prior study of a variety of human histiocytic neoplasms found that the mutational burden in one case of HS was higher than in other histiocytic tumors but that expression of PD-L1 in HS was the second lowest of all tumors, leading the authors to posit that HS patients may benefit from PD-1:PD-L1 blockade [13]. In canine HS, PD-L1 expression has been detected using IHC in 18/20 cases [14]. However, while *PDCD1* was identified as a DEG in two of three analyses in our data set, neither *CD274* (PD-L1) nor *PDCD1LG2* (PD-L2) were differentially expressed. Assessment of anti-PD-1 therapy in human HS patients revealed a favorable response in one patient, a transient response in another, and no benefit in a third patient [15–17]. While these cases suggest that PD-1 blockade alone will not be sufficient to control HS in all human patients, these mixed responses support future studies in which PD-1 blockade is assessed in combination with other therapeutics. Although we attempted to quantify PD-1 expression in canine HS with IHC, we were unable to obtain reliable staining of FFPE sections highlighting the need for the ongoing development of reagents for comparative oncology. Nonetheless, as the FDA has conditionally approved the first canine PD-1 blocking antibody, gilvetmab, canine HS now serves as a unique platform to systematically appraise the pre-clinical activity of PD-1 blockade, both alone and in combination with other therapeutics.

*TXNIP* encoding the tumor suppressor TXNIP was the most significant negative DEG in our dataset and was also decreased in human HS [8]*.* Intriguingly, DCs isolated from *Txnip*-deficient mice exhibit defective T cell activation, supporting decreased *TXNIP* expression as a putative mechanism of immune evasion in canine and human HS [18]. Finally, our transcriptomic and immunohistochemical datasets implicated a pro-tumorigenic role for osteopontin in canine and human HS. Osteopontin can suppress anti-tumor T cell function in the setting of glioma and colorectal cancer, supporting its function as an immune checkpoint and, therefore, a candidate immune suppressor in HS [5, 6].

It is worth noting that whilst we found good conservation of enriched and depleted DEGs between canine and human HS, there was a minority of DEGs that did not follow this pattern. One explanation could be due to interspecies differences. Alternatively, in our study, we used grossly normal pulmonary and splenic tissue as controls as opposed to reactive histiocytic tissues utilized by Egan et al. [8] which may also account for these discrepancies*.* Although reactive histiocytic processes are rarely reported in the dog, they most frequently affect skin and lymph nodes, and such tissues are not uniformly effaced [3, 19]. As such, we selected corresponding healthy tissues to the primary tumors for comparison, as non-histiocytic cells originating from organs unrelated to the tumor sites could confound our analyses.

As the two tumor sets we compared were from different tissue origins, this may have also impacted expression profiles. It is notable that non-supervised clustering of our dataset gave a clear segregation between tumors and normal tissues, however, there was no distinct clustering of the canine tumors based on tumor location. In work performed by Egan et al*.* [8] two distinct molecular subgroups of human HS were described. One HS subset was defined by alterations in *NF1* and/or *PTPN11* and frequently presented within the gastrointestinal (GI) tract, however their subsequent analyses found that gene clustering was not related to anatomic site [8]. Our prior work comparing T cell counts and gene expression between pulmonary and splenic HS implicated transcriptional changes relating to T cell inflammation and antigen presentation as major differences between pulmonary and splenic HS and provided rationale for investigating potential immune suppressors in this subset of canine tumors [4]. Whilst none of our reported canine HS cases were of GI origin, subsequent prospective studies using single cell profiling from fresh HS as well as reactive histiocytic tissues in greater numbers of dogs could be employed to validate the findings of our current approach. These studies will be designed to ensure that adequate tissues are banked to enable confirmation of transcriptomic findings using technologies such as qPCR and flow cytometry for PD-1 expression, which were precluded from our current studies due to the limited availability of stored clinical FFPE tissues for analyses.

Lack of model systems are a major challenge facing cancer immunotherapy, and this is amplified for rare diseases such as HS [20]. Evaluating anti-PD-1 therapy in canine HS could further support or refute the preclinical value of this approach. Moreover, such studies may also reveal the effect of targeting subsets of HS patients based on additional parameters, including TIL density and PD-1 expression, both of which predict response to PD-1 blockade in other tumors such as melanoma [21]. Finally, lack of consistent responses to PD-1 blockade may also indicate that alternative immune evasive candidates, including those identified here, should be pursued. Strategies to target TXNIP and osteopontin are being developed and such approaches could be appraised alone or in combination with other therapies in canine HS [6, 22], thus paving the way to improve treatment of human HS, which remains a deadly disease.

## Supplementary Information

Below is the link to the electronic supplementary material.

Supplementary file1 (PDF 1729 kb)

## Data Availability

Data is available upon reasonable request to the corresponding author.
