# Supplementary material for: Identification of immune suppressor candidates utilizing comparative transcriptional profiling in histiocytic sarcoma
Source: Cancer Immunol Immunother. 2025 Jan 3;74(2):61. doi: 10.1007/s00262-024-03908-x (PMC11699166; doi:10.1007/s00262-024-03908-x)
Supplement: Supplementary file 1 — Supplementary file1 (PDF 1729 kb) [file 262_2024_3908_MOESM1_ESM.pdf]

Supplementary Materials and methods

Western blotting

5µg of boiled recombinant canine osteopontin (Creative Biomart, SPP1-7901D) protein per lane was loaded onto a 7.5% acrylamide running gel prior to electrophoresis. Samples were then transferred from the acrylamide gel to a PVDF membrane via wet transfer.

The PVDF membrane was then blocked with 5% milk powder in tris-buffered saline with Tween 20 (TBST) for 1 hour followed by washing with TBST. The membrane was incubated overnight with 1:1000 primary anti-osteopontin antibody (Rockland Immunochemicals) in 1% bovine serum albumin (BSA) in TBST with shaking and was subsequently washed three times in TBST Subsequent incubation with 1:10,000 secondary anti-rabbit horseradish peroxidase (Cell Signaling) in 1% BSA in TBST with shaking was performed. Membranes were imaged with an Amersham Imager 680 (General Electric Life Sciences) after incubating membrane with ECL substrate using Clarity Western ECL kit (Bio-Rad Laboratories).

Figure S1. Differentially expressed genes in canine pulmonary histiocytic sarcoma (HS). Immune profiling was performed on canine tissues using the NanoString nCounter Canine IO Panel and data were analyzed using ROSALIND® platform. Heatmap of genes differentially expressed between three canine pulmonary HS tumors and three normal lungs ($p \leq 0.01$, fold change ≥ 1.5 or ≤ -1.5).

Figure S2. Differentially expressed genes in canine splenic histiocytic sarcoma (HS). Immune profiling was performed on canine tissues using the NanoString nCounter Canine IO Panel and data were analyzed using ROSALIND® platform. Heatmap of genes differentially expressed between three canine splenic HS tumors and three normal spleens ($p \leq 0.01$, fold change ≥ 1.5 or ≤ -1.5).

Figure S3. *PDCD1* expression is positively associated with survival time in canine HS. Immune profiling was performed on canine tissues using the NanoString nCounter Canine IO Panel and normalized expression values were obtained using ROSALIND® platform and plotted against tumor-specific survival times in six dogs diagnosed with HS. Spearmen correlation performed using Prism 10 (GraphPad Software) with Spearman's rho (r_s) and two-tailed p value reported.

Figure S4. Demonstration of cross reactivity of an antibody against canine osteopontin. Western blots were performed on recombinant canine osteopontin using a commercially available polyclonal rabbit antibody raised against a synthetic peptide from human osteopontin. 5μg of recombinant canine osteopontin was loaded in lanes 1 and 2 revealing a predominant

band with an estimated mass slightly below 40KDa consistent with SDS-PAGE data from the protein manufacturer. A molecular mass ladder (Thermo Scientific Page Ruler Prestained Protein Ladder) with corresponding mass units in KDa is present on the left side of the gel.

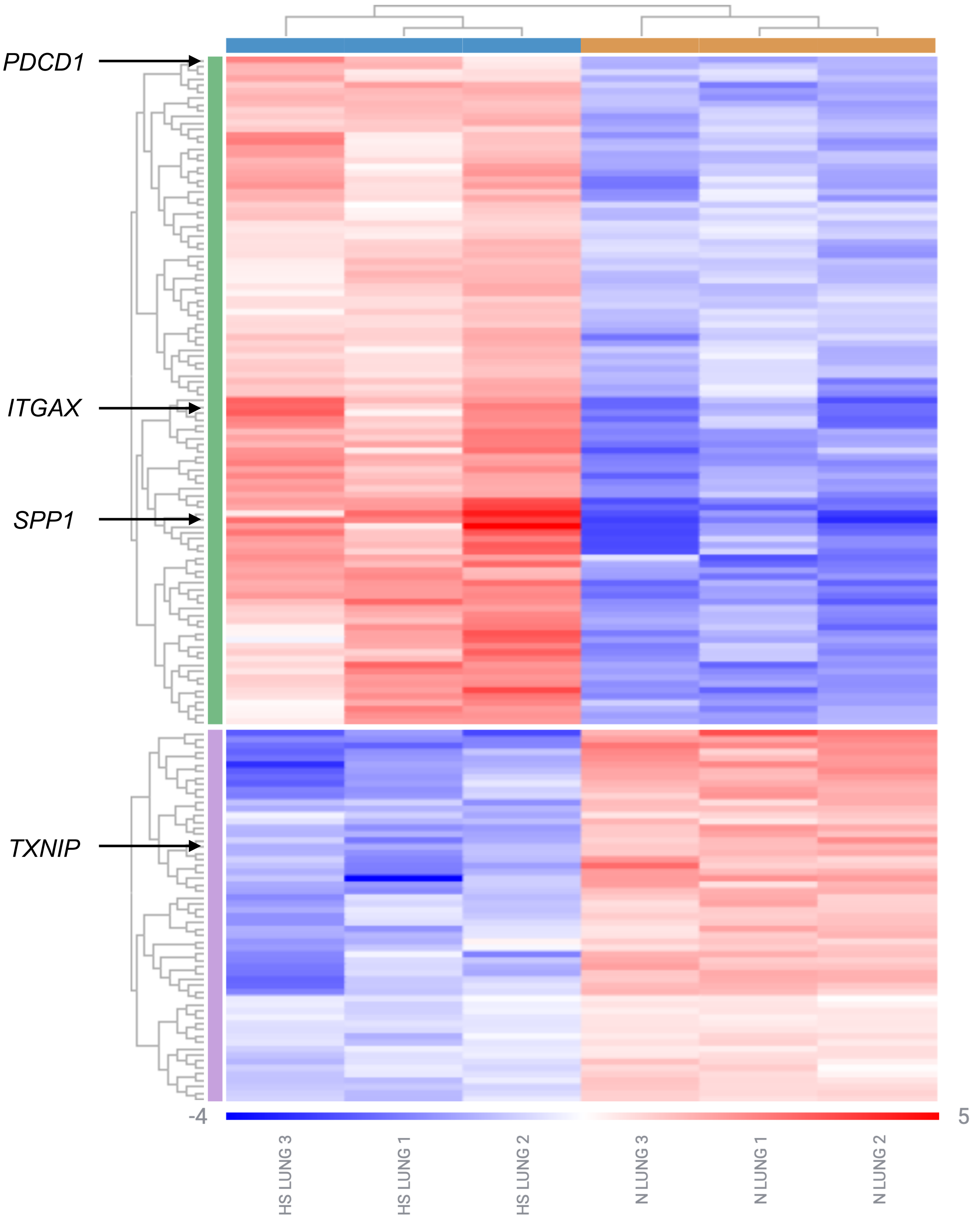

PDCD1
ITGAX
SPP1
TXNIP
-4
5
HS LUNG 3
HS LUNG 1
HS LUNG 2
N LUNG 3
N LUNG 1
N LUNG 2

Figure S1

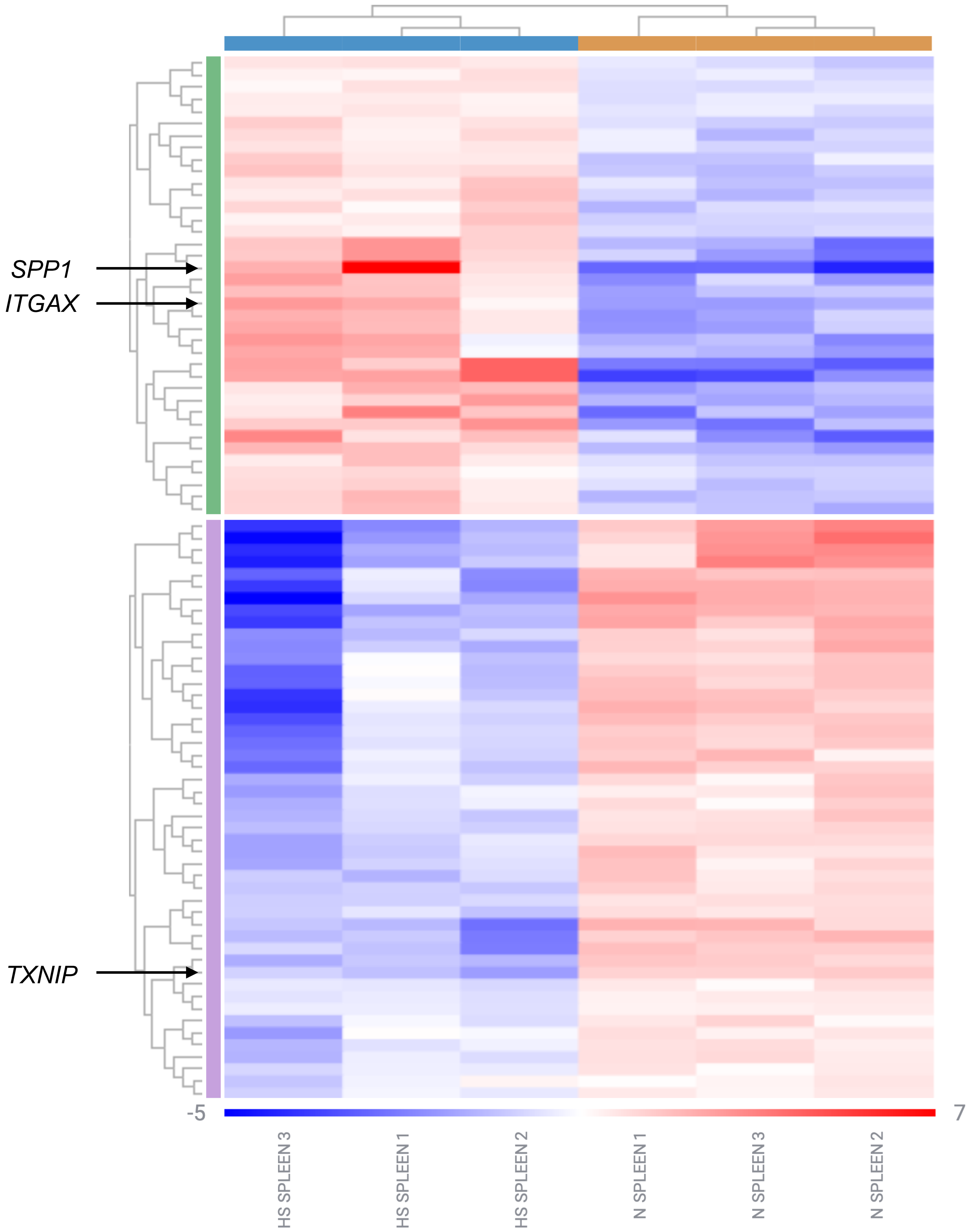
SPP1
ITGAX
TXNIP
-5
7
HS SPLEEN 3
HS SPLEEN 1
HS SPLEEN 2
N SPLEEN 1
N SPLEEN 3
N SPLEEN 2

Figure S2

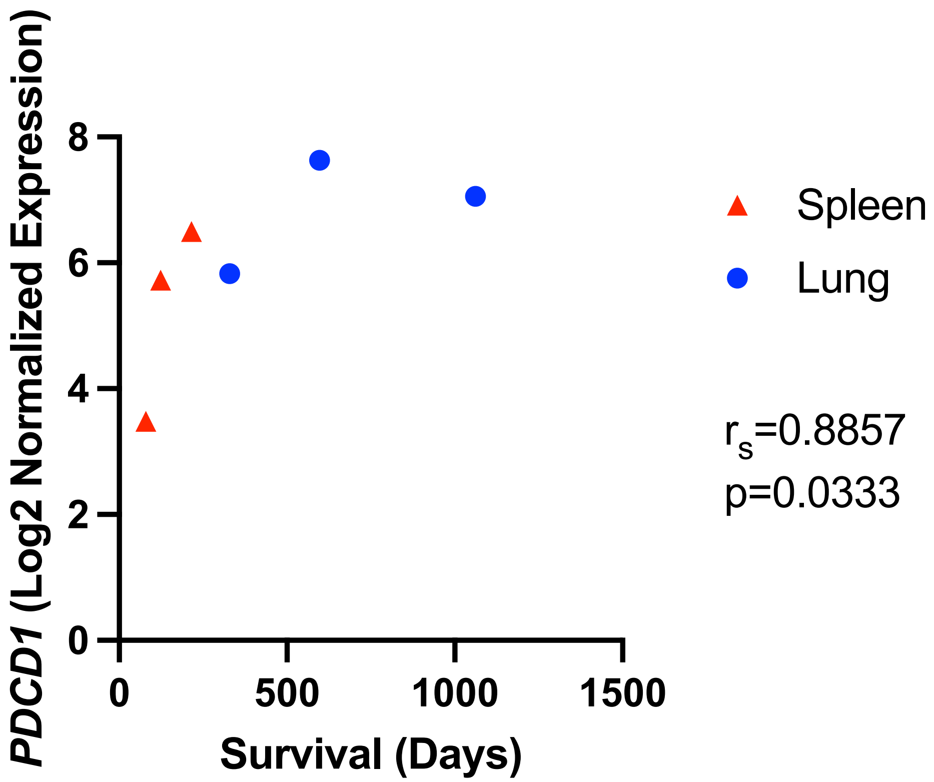
PDCD1 (Log2 Normalized Expression)
8
6
4
2
0
0
500
1000
1500
Survival (Days)
Spleen
Lung
r_s=0.8857
p=0.0333

Figure S3

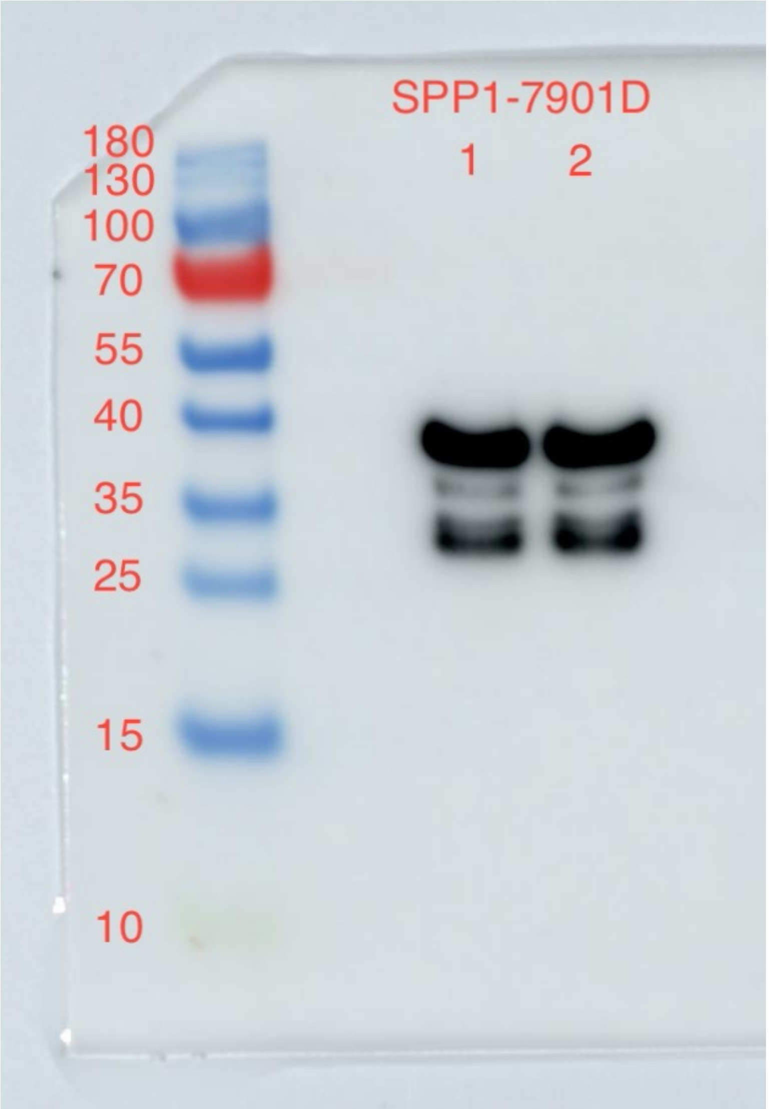

Figure S4

Table S1 Characteristics of canine tissues used for transcriptional profiling.

Sample ID	Tissue type	Signalment	*CD3+ TIL density/mm^2	*Survival time (days)
HS LUNG 1	Pulmonary HS	6-year-old male neutered greyhound	5865	1062
HS LUNG 2	Pulmonary HS	9-year-old male neutered Portuguese water dog	5105	329
HS LUNG 3	Pulmonary HS	4-year-old female spayed Bernese mountain dog	3017	597
HS SPLEEN 1	Splenic HS	8-year-old male neutered golden retriever	1929	215
HS SPLEEN 2	Splenic HS	11-year-old male standard poodle	273	124
HS SPLEEN 3	Splenic HS	11-year-old male neutered Rottweiler	184	80
N LUNG 1	Control lung	10-year-old female spayed boxer	N/A	N/A
N SPLEEN 1	Control spleen		N/A	N/A
N LUNG 2	Control lung	14-year-old male castrated mix breed	N/A	N/A
N SPLEEN 2	Control spleen		N/A	N/A
N LUNG 3	Control lung	8-year-old male castrated boxer	N/A	N/A
N SPLEEN 3	Control spleen		N/A	N/A

*These data were originally published by Lenz *et al.* CII 2022.

Table S2 Differentially expressed genes between all canine HS samples and all control tissue

Name	Description	Fold Change	Log Fold Change	p-Value	p-Adj
**BCL2**	B-cell CLL/lymphoma 2	-1.85938	-0.894818	0.003252	0.028533
**BRCA1**	breast cancer 1, early onset	3.84678	1.94365	0.000998	0.0137
**CCL5**	chemokine (C-C motif) ligand 5	13.4292	3.7473	0.000282	0.006965
**BAX**	BCL2-associated X protein	2.33275	1.22203	2.61E-05	0.002292
**IL15**	interleukin 15	2.5215	1.33428	0.00523	0.037239
**IL10**	interleukin 10	2.07237	1.05128	0.004401	0.032857
**COL1A1**	collagen, type I, alpha 1	16.7058	4.06228	2.84E-05	0.002292
**CD80**	CD80 molecule	2.68474	1.42478	3.58E-05	0.002292
**ITGB2**	integrin, beta 2 (complement component 3 receptor 3 and 4 subunit)	5.66977	2.50329	0.004236	0.032538
**ITGA2B**	integrin, alpha 2b (platelet glycoprotein IIb of IIb/IIIa complex, antigen CD41)	-10.3333	-3.36923	0.001414	0.017831
**IFNG**	interferon gamma	10.8334	3.43741	0.003902	0.030659
**KIT**	v-kit Hardy-Zuckerman 4 feline sarcoma viral oncogene homolog	-8.49998	-3.08746	2.5E-05	0.002292
**CXCL8**	interleukin 8	-8.36004	-3.06351	0.004649	0.03405
**MAPK14**	mitogen-activated protein kinase 14	-2.02093	-1.01502	0.003864	0.030659
**EGFR**	epidermal growth factor receptor	-3.15719	-1.65864	0.008101	0.047195
**CDH1**	cadherin 1, type 1, E-cadherin (epithelial)	-7.15633	-2.83922	0.003911	0.030659
**PDGFRA**	platelet-derived growth factor receptor, alpha polypeptide	-5.96272	-2.57597	0.001942	0.02213
**CAMP**	cathelicidin antimicrobial peptide	-22.7619	-4.50855	0.002364	0.0249
**IGF1R**	insulin-like growth factor 1 receptor	-4.38556	-2.13276	0.000743	0.011007
**JAK1**	Janus kinase 1	-1.56516	-0.646308	0.000633	0.009921
**CLU**	clusterin	3.09852	1.63158	0.00739	0.044504
**CCL4**	chemokine (C-C motif) ligand 4	5.28246	2.40121	0.003863	0.030659
**CCL3**	chemokine (C-C motif) ligand 3	7.22596	2.85319	0.000139	0.004847
**CCL21**	chemokine (C-C motif) ligand 21	-9.48863	-3.2462	0.002312	0.024697
**TLR2**	toll-like receptor 2	3.24849	1.69977	0.000168	0.005604
**CXCL12**	chemokine (C-X-C motif) ligand 12	-6.74468	-2.75375	0.006591	0.041509
**BRCA2**	breast cancer 2, early onset	2.74233	1.4554	0.001216	0.015846
**RPS6**	ribosomal protein S6	-2.15366	-1.10679	0.003741	0.030608
**ARG1**	arginase 1	2.65117	1.40663	0.001964	0.02213
**DLA-64**	MHC class I DLA-64	2.70298	1.43455	0.004274	0.032538
**C2**	complement component 2	3.09136	1.62824	0.000178	0.00571
**FYN**	FYN oncogene related to SRC, FGR, YES	-2.25876	-1.17553	0.004598	0.033998
**PTK2**	protein tyrosine kinase 2	-2.76793	-1.46881	0.002959	0.028034
**TXNIP**	thioredoxin interacting protein	-5.93616	-2.56953	1.6E-08	1.23E-05
**SERPING1**	serpin peptidase inhibitor, clade G (C1 inhibitor), member 1	-3.28741	-1.71695	0.000345	0.007161
**ATG7**	autophagy related 7	1.91006	0.933619	0.002805	0.026961
**IL17RB**	interleukin 17 receptor B	-4.12931	-2.0459	0.005464	0.038198

**SMARCA4**	SWI/SNF related, matrix associated, actin dependent regulator of chromatin, subfamily a, member 4	2.07867	1.05566	0.000228	0.006745
**RB1**	retinoblastoma 1	-1.87907	-0.910022	0.003567	0.029493
**MME**	membrane metallo-endopeptidase	-4.63192	-2.21161	0.002554	0.025843
**KRT18**	keratin 18	-6.9958	-2.80649	0.000828	0.012018
**MAPK8**	mitogen-activated protein kinase 8	-2.25018	-1.17004	7.16E-05	0.003301
**NRP1**	neuropilin 1	-2.86594	-1.51901	2.61E-05	0.002292
**MAP3K1**	mitogen-activated protein kinase kinase kinase 1, E3 ubiquitin protein ligase	-1.87751	-0.908821	0.005787	0.038764
**SPP1**	secreted phosphoprotein 1	113.461	6.82605	6.54E-06	0.001257
**ATF2**	activating transcription factor 2	-1.96005	-0.97089	0.000645	0.009921
**IKBKE**	inhibitor of kappa light polypeptide gene enhancer in B-cells, kinase epsilon	3.01323	1.59131	0.000277	0.006965
**IDH2**	isocitrate dehydrogenase 2 (NADP+), mitochondrial	2.9856	1.57802	0.000434	0.008226
**TNFRSF14**	tumor necrosis factor receptor superfamily, member 14	2.57268	1.36327	0.001553	0.019268
**NFATC3**	nuclear factor of activated T-cells, cytoplasmic, calcineurin-dependent 3	-1.56508	-0.646239	0.005619	0.038238
**CREBBP**	CREB binding protein	-1.80419	-0.85135	3.87E-05	0.002292
**ICAM2**	intercellular adhesion molecule 2	-3.02877	-1.59873	0.000299	0.006965
**CCL14**	chemokine (C-C motif) ligand 14	-10.7397	-3.42488	0.007329	0.044504
**ERBB3**	v-erb-b2 avian erythroblastic leukemia viral oncogene homolog 3	-4.3806	-2.13113	0.003485	0.029131
**CDK4**	cyclin-dependent kinase 4	1.66097	0.732022	0.002803	0.026961
**PRDM1**	PR domain containing 1, with ZNF domain	4.65477	2.21871	0.006639	0.041509
**PIK3CG**	phosphatidylinositol-4,5-bisphosphate 3-kinase, catalytic subunit gamma	3.4305	1.77842	0.000614	0.00983
**TOLLIP**	toll interacting protein	2.57614	1.36521	9.15E-05	0.003705
**JAK2**	Janus kinase 2	1.60792	0.685194	0.003053	0.028287
**LOC484306**	leukocyte immunoglobulin-like receptor, subfamily B (with TM and ITIM domains), member 5-like	5.37699	2.4268	8.31E-05	0.003551
**LTF**	lactotransferrin	-6.55952	-2.71359	0.006249	0.040045
**CCR5**	chemokine (C-C motif) receptor 5	5.4527	2.44697	0.000496	0.008479
**CCR1**	chemokine (C-C motif) receptor 1	3.23046	1.69174	0.000972	0.013586
**FCER2**	Fc fragment of IgE, low affinity II, receptor for (CD23)	-7.38204	-2.88402	0.000472	0.008316
**CD70**	CD70 molecule	3.18751	1.67243	0.002777	0.026961
**SPRY2**	sprouty homolog 2 (Drosophila)	-1.85577	-0.892017	0.001765	0.021546
**TNFSF13B**	tumor necrosis factor (ligand) superfamily, member 13b	2.60948	1.38376	0.003265	0.028533
**CPA3**	carboxypeptidase A3 (mast cell)	-6.75783	-2.75656	0.007869	0.046194
**SRC**	v-src sarcoma (Schmidt-Ruppin A-2) viral oncogene homolog (avian)	1.76735	0.821585	0.005518	0.038231
**LBP**	lipopolysaccharide binding protein	-8.05659	-3.01017	0.004336	0.03269
**PDCD1**	programmed cell death 1	3.99152	1.99694	0.003947	0.030659
**CHEK2**	checkpoint kinase 2	2.50747	1.32623	0.002517	0.025808
**IRAK4**	interleukin-1 receptor-associated kinase 4	1.78668	0.837284	0.000374	0.007568
**C3AR1**	complement component 3a receptor 1	3.02788	1.59831	0.007805	0.046167

**PIK3R1**	phosphoinositide-3-kinase, regulatory subunit 1 (alpha)	-1.95971	-0.970638	0.007408	0.044504
**NPNT**	nephronectin	-9.80488	-3.2935	0.000298	0.006965
**BCL6**	B-cell CLL/lymphoma 6	-1.97636	-0.982845	0.008238	0.047632
**TLR5**	toll-like receptor 5	6.75001	2.75489	0.007332	0.044504
**CD84**	CD84 molecule	6.45989	2.69151	0.00749	0.044651
**FGFR3**	fibroblast growth factor receptor 3	-7.0606	-2.81979	0.000269	0.006965
**PRF1**	perforin 1 (pore forming protein)	5.22953	2.38668	0.000449	0.008228
**DUSP1**	dual specificity phosphatase 1	-4.37305	-2.12864	0.000323	0.00709
**IL17B**	interleukin 17B	-3.17708	-1.6677	0.005133	0.03689
**ITGA1**	integrin, alpha 1	-4.67087	-2.22369	3.76E-05	0.002292
**MCAM**	melanoma cell adhesion molecule	-3.72988	-1.89913	0.00229	0.024697
**C1QBP**	complement component 1, q subcomponent binding protein	2.55937	1.35579	0.001946	0.02213
**CD68**	CD68 molecule	4.96778	2.3126	0.00197	0.02213
**IL12RB2**	interleukin 12 receptor, beta 2	-7.91665	-2.98489	0.005389	0.038022
**CDH5**	cadherin 5, type 2 (vascular endothelium)	-13.8305	-3.78978	0.000315	0.00709
**LFNG**	LFNG O-fucosylpeptide 3-beta-N-acetylglucosaminyltransferase	2.29482	1.19838	0.000602	0.00983
**ITGAX**	integrin, alpha X (complement component 3 receptor 4 subunit)	11.4743	3.52033	8.5E-07	0.000218
**IL4R**	interleukin 4 receptor	2.12395	1.08675	0.000409	0.00806
**S100A8**	S100 calcium binding protein A8	-7.42042	-2.8915	0.003308	0.028585
**S100A9**	S100 calcium binding protein A9	-6.98979	-2.80525	0.006592	0.041509
**NLRP3**	NLR family, pyrin domain containing 3	4.11052	2.03932	0.000237	0.006745
**TRAF3**	TNF receptor-associated factor 3	2.08443	1.05965	0.00228	0.024697
**CCL7**	chemokine (C-C motif) ligand 7	9.02596	3.17408	0.001983	0.02213
**CYBB**	cytochrome b-245, beta polypeptide (chronic granulomatous disease)	5.2709	2.39805	0.005797	0.038764
**CXCR3**	chemokine (C-X-C motif) receptor 3	7.46752	2.90063	6.41E-05	0.003301
**IRAK1**	interleukin-1 receptor-associated kinase 1	2.88236	1.52725	0.006247	0.040045
**MIF**	macrophage migration inhibitory factor	3.80145	1.92655	0.000744	0.011007
**CD14**	CD14 molecule	6.15223	2.62111	0.000123	0.004507
**SHMT2**	serine hydroxymethyltransferase 2 (mitochondrial)	4.97984	2.3161	1.77E-05	0.002292
**BCR**	breakpoint cluster region	-4.38109	-2.13129	2.91E-07	0.000112
**CARD9**	caspase recruitment domain family, member 9	2.80768	1.48938	0.00141	0.017831
**CREB1**	cAMP responsive element binding protein 1	-2.12135	-1.08498	0.000252	0.006921
**IL21R**	interleukin 21 receptor	4.3722	2.12836	0.001184	0.0157
**TANK**	TRAF family member-associated NFKB activator	1.93778	0.954408	0.003407	0.029114
**TREM2**	triggering receptor expressed on myeloid cells 2	17.7803	4.15221	0.000207	0.00638
**TNFSF11**	tumor necrosis factor (ligand) superfamily, member 11	21.3837	4.41844	0.003473	0.029131
**CXCL14**	chemokine (C-X-C motif) ligand 14	4.46231	2.15779	0.000593	0.00983
**LGALS1**	lectin, galactoside-binding, soluble, 1	2.6556	1.40904	0.000476	0.008316
**LY96**	lymphocyte antigen 96	2.61201	1.38516	0.005905	0.039147
**PECAM1**	platelet/endothelial cell adhesion molecule 1	-11.1743	-3.48211	3.88E-05	0.002292

**CLEC7A**	C-type lectin domain family 7, member A	7.05952	2.81957	0.000111	0.004266
**CD247**	CD247 molecule	-14.5	-3.85798	0.000338	0.007161
**CD3EAP**	CD3e molecule, epsilon associated protein	2.6129	1.38565	0.005585	0.038238
**TRAF1**	TNF receptor-associated factor 1	2.20199	1.13881	0.002635	0.026315
**IL7**	interleukin 7	-8.70588	-3.12199	0.00092	0.013096
**CDKN2A**	cyclin-dependent kinase inhibitor 2A (melanoma, p16, inhibits CDK4)	6.66392	2.73637	6.81E-05	0.003301
**CEBPA**	CCAAT/enhancer binding protein (C/EBP), alpha	4.38778	2.13349	7.3E-05	0.003301
**S100A12**	S100 calcium binding protein A12	-11.1226	-3.47542	0.003222	0.028533
**TLR6**	toll-like receptor 6	3.03726	1.60277	0.002436	0.025316
**SIGIRR**	single immunoglobulin and toll-interleukin 1 receptor (TIR) domain	2.52941	1.3388	0.003098	0.028364
**LY9**	lymphocyte antigen 9	2.49191	1.31725	0.006119	0.039877
**IRF1**	interferon regulatory factor 1	3.89964	1.96334	0.002989	0.028034
**MAPK3**	mitogen-activated protein kinase 3	-2.16165	-1.11213	0.003141	0.028415
**PYCARD**	PYD and CARD domain containing	2.90378	1.53793	0.004896	0.035519
**LOC102156836**	uncharacterized LOC102156836	-17.8228	-4.15565	0.001088	0.014675
**IGHM**		-13.1725	-3.71946	0.007026	0.043571
**LOC100856270**		4.34854	2.12053	0.000439	0.008226

Table S3 Differentially expressed genes between canine pulmonary HS and normal lung

Name	Description	Fold Change	Log Fold Change	p-Value
**CD8A**	CD8a molecule	24.0299	4.58676	0.000795
**CTSS**	cathepsin S	4.31252	2.10853	0.007311
**TERT**	telomerase reverse transcriptase	1.8595	0.894915	0.008759
**IL2RB**	interleukin 2 receptor, beta	7.05086	2.8178	0.003970
**CCL5**	chemokine (C-C motif) ligand 5	29.7845	4.89649	0.003814
**BAX**	BCL2-associated X protein	2.75217	1.46057	0.001467
**IL15**	interleukin 15	3.35627	1.74686	0.007435
**BCL2L1**	BCL2-like 1	-1.80263	-0.850106	0.006877
**IL13RA1**	interleukin 13 receptor, alpha 1	-7.36084	-2.87987	0.000895
**COL1A1**	collagen, type I, alpha 1	8.2462	3.04373	0.007434
**CTLA4**	cytotoxic T-lymphocyte-associated protein 4	7.51926	2.91059	0.005536
**CD80**	CD80 molecule	2.65921	1.411	0.009574
**ITGB2**	integrin, beta 2 (complement component 3 receptor 3 and 4 subunit)	8.45597	3.07997	0.004113
**IL1A**	interleukin 1, alpha	3.13015	1.64623	0.002771
**KIT**	v-kit Hardy-Zuckerman 4 feline sarcoma viral oncogene homolog	-8.13634	-3.02438	0.001892
**IL2RG**	interleukin 2 receptor, gamma	12.5463	3.64919	0.002864
**ERBB2**	v-erb-b2 erythroblastic leukemia viral oncogene homolog 2, neuro/glioblastoma derived oncogene homolog (avian)	-3.12001	-1.64155	0.009585
**MMP9**	matrix metallopeptidase 9 (gelatinase B, 92kDa gelatinase, 92kDa type IV collagenase)	48.3157	5.59442	0.007518
**PLA2G1B**	phospholipase A2, group IB (pancreas)	-38.8462	-5.2797	0.004789
**EGFR**	epidermal growth factor receptor	-3.87114	-1.95276	0.004088
**CDH1**	cadherin 1, type 1, E-cadherin (epithelial)	-6.12959	-2.61579	0.006217
**PDGFRA**	platelet-derived growth factor receptor, alpha polypeptide	-5.2443	-2.39075	0.005439
**IGF1R**	insulin-like growth factor 1 receptor	-5.80425	-2.53711	0.003219
**FASLG**	Fas ligand (TNF superfamily, member 6)	5.99999	2.58496	0.001901
**CD3E**	CD3e molecule, epsilon (CD3-TCR complex)	8.49998	3.08746	0.008185
**CCL4**	chemokine (C-C motif) ligand 4	12.9667	3.69674	0.009894
**CCL3**	chemokine (C-C motif) ligand 3	13.4694	3.75161	0.001497
**CCL21**	chemokine (C-C motif) ligand 21	-10.3698	-3.37431	0.001612
**TLR2**	toll-like receptor 2	3.16778	1.66347	0.007113
**BRCA2**	breast cancer 2, early onset	3.2037	1.67974	0.007277
**DLA-64**	MHC class I DLA-64	5.15517	2.36602	0.006085
**C2**	complement component 2	3.9805	1.99295	0.001570
**PSMB9**	proteasome (prosome, macropain) subunit, beta type, 9 (large multifunctional peptidase 2)	2.22868	1.15619	0.001004
**CDKN1A**	cyclin-dependent kinase inhibitor 1A (p21, Cip1)	-3.24858	-1.69981	0.002812
**PTK2**	protein tyrosine kinase 2	-4.7479	-2.24729	0.008483
**CDKN2C**	cyclin-dependent kinase inhibitor 2C (p18, inhibits CDK4)	3.13158	1.64689	0.004023

**TXNIP**	thioredoxin interacting protein	-6.41692	-2.68188	0.000296
**S100A10**	S100 calcium binding protein A10	-3.82532	-1.93558	0.003499
**CD36**	CD36 molecule (thrombospondin receptor)	-5.30918	-2.40849	0.000219
**SERPING1**	serpin peptidase inhibitor, clade G (C1 inhibitor), member 1	-4.19107	-2.06732	0.005035
**ANXA1**	annexin A1	-5.66101	-2.50106	0.000280
**SETD2**	SET domain containing 2	-2.80556	-1.48829	0.002809
**SMARCA4**	SWI/SNF related, matrix associated, actin dependent regulator of chromatin, subfamily a, member 4	2.36289	1.24055	0.008764
**RB1**	retinoblastoma 1	-1.94773	-0.961796	3.02E-05
**MME**	membrane metallo-endopeptidase	-2.65156	-1.40684	0.004873
**MAPK1**	mitogen-activated protein kinase 1	-5.32544	-2.4129	8.88E-07
**KRT18**	keratin 18	-8.15582	-3.02783	0.000367
**KRT7**	keratin 7	-6.43384	-2.68568	0.003170
**NRP1**	neuropilin 1	-3.41328	-1.77116	0.003070
**MAP2K1**	mitogen-activated protein kinase kinase 1	-2.29425	-1.19802	0.002123
**SPP1**	secreted phosphoprotein 1	33.5963	5.07023	0.000227
**CFI**	complement factor I	-6.79183	-2.7638	0.002890
**ATF2**	activating transcription factor 2	-3.09119	-1.62816	0.000165
**IKBKE**	inhibitor of kappa light polypeptide gene enhancer in B-cells, kinase epsilon	3.42001	1.774	0.009468
**CD3D**	CD3d molecule, delta (CD3-TCR complex)	8.85916	3.14717	0.000978
**TNFRSF14**	tumor necrosis factor receptor superfamily, member 14	4.62364	2.20903	0.007218
**CREBBP**	CREB binding protein	-1.79961	-0.847683	0.001958
**ERBB3**	v-erb-b2 avian erythroblastic leukemia viral oncogene homolog 3	-4.80122	-2.2634	0.004736
**NCF4**	neutrophil cytosolic factor 4, 40kDa	3.31183	1.72763	0.009010
**EPCAM**	epithelial cell adhesion molecule	-5.17142	-2.37056	0.009989
**EGR1**	early growth response 1	-8.6849	-3.11851	0.002326
**LTB**	lymphotoxin beta (TNF superfamily, member 3)	14.0	3.80735	0.001209
**NDRG1**	N-myc downstream regulated 1	-2.4166	-1.27298	0.001899
**KDR**	kinase insert domain receptor (a type III receptor tyrosine kinase)	-2.79743	-1.4841	0.009328
**NOD1**	nucleotide-binding oligomerization domain containing 1	-1.677	-0.745883	0.008346
**VEGFC**	vascular endothelial growth factor C	-3.5837	-1.84145	0.004813
**CD8B**	CD8b molecule	7.30189	2.86827	0.005341
**PIK3CG**	phosphatidylinositol-4,5-bisphosphate 3-kinase, catalytic subunit gamma	6.94806	2.79661	0.001671
**DLA-79**	MHC class Ib	6.03373	2.59305	0.000636
**NR1H3**	nuclear receptor subfamily 1, group H, member 3	17.6627	4.14263	0.001395
**TOLLIP**	toll interacting protein	3.53591	1.82208	0.002549
**SYK**	spleen tyrosine kinase	2.54907	1.34997	0.001701
**LAIR1**	leukocyte-associated immunoglobulin-like receptor 1	3.74467	1.90484	0.009889
**GSK3A**	glycogen synthase kinase 3 alpha	-1.98588	-0.989778	6.96E-05
**CD79A**	CD79a molecule, immunoglobulin-associated alpha	11.2	3.48543	0.007087
**IRAK2**	interleukin-1 receptor-associated kinase 2	-4.92301	-2.29954	1.58E-05

**BHLHE40**	basic helix-loop-helix family, member e40	-5.19902	-2.37824	0.001838
**CCR5**	chemokine (C-C motif) receptor 5	13.1482	3.71679	0.000977
**IL12RB1**	interleukin 12 receptor, beta 1	5.94555	2.57181	0.000948
**EBI3**	Epstein-Barr virus induced 3	5.35795	2.42168	0.006996
**TNFSF13B**	tumor necrosis factor (ligand) superfamily, member 13b	4.61538	2.20645	0.003263
**EOMES**	eomesodermin	6.23881	2.64127	0.003508
**HCK**	hemopoietic cell kinase	1.952	0.964952	0.009600
**PDCD1**	programmed cell death 1	6.43183	2.68523	0.008445
**CHEK2**	checkpoint kinase 2	4.01514	2.00545	0.005223
**ANKRD22**	ankyrin repeat domain 22	9.58335	3.26053	0.001651
**ITGA5**	integrin, alpha 5 (fibronectin receptor, alpha polypeptide)	-5.20771	-2.38065	0.003208
**IRAK4**	interleukin-1 receptor-associated kinase 4	2.14111	1.09836	0.003561
**IL15RA**	interleukin 15 receptor, alpha	9.29168	3.21594	0.007952
**CX3CL1**	chemokine (C-X3-C motif) ligand 1	-7.23809	-2.85561	0.006512
**NLRC5**	NLR family, CARD domain containing 5	3.51046	1.81166	0.002877
**C1QB**	complement component 1, q subcomponent, B chain	10.4188	3.38112	0.000136
**TNFRSF8**	tumor necrosis factor receptor superfamily, member 8	14.0	3.80735	0.002987
**THBS1**	thrombospondin 1	-7.7231	-2.94918	0.004593
**NPNT**	nephronectin	-12.645	-3.66049	3.9E-05
**CD96**	CD96 molecule	9.68418	3.27563	0.009457
**TIGIT**	T cell immunoreceptor with Ig and ITIM domains	16.8823	4.07744	0.004334
**BCL6**	B-cell CLL/lymphoma 6	-2.46268	-1.30023	0.004349
**ERBB4**	v-erb-b2 avian erythroblastic leukemia viral oncogene homolog 4	-14.8777	-3.89508	2.61E-05
**TLR5**	toll-like receptor 5	8.90546	3.15469	0.005070
**CD84**	CD84 molecule	23.2	4.53605	0.000754
**CD48**	CD48 molecule	9.03729	3.17589	0.004513
**FGFR3**	fibroblast growth factor receptor 3	-13.8	-3.7866	0.002238
**PRF1**	perforin 1 (pore forming protein)	9.71794	3.28065	0.001744
**DUSP1**	dual specificity phosphatase 1	-8.86844	-3.14868	0.000247
**CSF1R**	colony stimulating factor 1 receptor	6.13715	2.61757	0.004294
**ITGA1**	integrin, alpha 1	-4.4693	-2.16005	0.003554
**JAM3**	junctional adhesion molecule 3	-2.43851	-1.286	0.008058
**MCAM**	melanoma cell adhesion molecule	-5.55909	-2.47485	0.000250
**CD3G**	CD3g molecule, gamma (CD3-TCR complex)	9.76087	3.28701	0.001361
**CD68**	CD68 molecule	11.142	3.47793	0.004694
**PIK3CD**	phosphatidylinositol-4,5-bisphosphate 3-kinase, catalytic subunit delta	3.17483	1.66668	0.001412
**IRF8**	interferon regulatory factor 8	7.78059	2.95988	0.007082
**CDH5**	cadherin 5, type 2 (vascular endothelium)	-22.926	-4.51891	0.000130
**ITGAX**	integrin, alpha X (complement component 3 receptor 4 subunit)	13.3561	3.73943	0.001660
**CIITA**	class II, major histocompatibility complex, transactivator	3.93221	1.97534	0.005578

**MEFV**	Mediterranean fever	3.06061	1.61382	0.007757
**GBP5**	guanylate binding protein 5	4.13412	2.04758	0.002656
**LOC490356**	cytokine SCM-1 beta-like	2.98781	1.57909	0.008096
**NLRP3**	NLR family, pyrin domain containing 3	4.94736	2.30666	0.009183
**GZMB**	granzyme B (granzyme 2, cytotoxic T-lymphocyte-associated serine esterase 1)	5.75207	2.52408	0.008483
**TRAF3**	TNF receptor-associated factor 3	3.05713	1.61218	0.000774
**CXCR3**	chemokine (C-X-C motif) receptor 3	15.019	3.90872	0.000416
**DLA-12**	MHC class I DLA-12	3.03829	1.60326	0.005488
**MIF**	macrophage migration inhibitory factor	3.51141	1.81205	0.001707
**CD2**	CD2 molecule	6.98113	2.80346	0.004513
**SHMT2**	serine hydroxymethyltransferase 2 (mitochondrial)	6.53284	2.70771	0.000636
**BCR**	breakpoint cluster region	-5.48268	-2.45488	1.74E-05
**CXCL16**	chemokine (C-X-C motif) ligand 16	5.54059	2.47004	0.002872
**CNP**	2',3'-cyclic nucleotide 3' phosphodiesterase	2.54272	1.34637	0.006703
**DLA-DOB**	major histocompatibility complex, class II, DO beta	14.6385	3.8717	0.000787
**IL21R**	interleukin 21 receptor	11.8359	3.5651	0.000139
**CXCR6**	chemokine (C-X-C motif) receptor 6	8.96156	3.16375	0.002480
**PRKCE**	protein kinase C, epsilon	-1.82797	-0.870242	0.006232
**SLAMF7**	SLAM family member 7	3.60493	1.84997	0.006888
**LY96**	lymphocyte antigen 96	2.95154	1.56147	0.009962
**PECAM1**	platelet/endothelial cell adhesion molecule 1	-17.1701	-4.10183	1.74E-05
**KLRK1**	killer cell lectin-like receptor subfamily K, member 1	2.55825	1.35516	0.006519
**IL1RL1**	interleukin 1 receptor-like 1	-6.62904	-2.7288	0.005551
**CD27**	CD27 molecule	18.8572	4.23704	0.001816
**CD3EAP**	CD3e molecule, epsilon associated protein	2.43306	1.28277	0.005269
**CD5**	CD5 molecule	7.69451	2.94383	0.000587
**CEACAM1**	carcinoembryonic antigen-related cell adhesion molecule 1	4.23679	2.08297	0.000474
**IL7R**	interleukin 7 receptor	4.19607	2.06904	0.005318
**TRAF1**	TNF receptor-associated factor 1	2.72697	1.4473	0.001252
**SH2D1A**	SH2 domain containing 1A	17.027	4.08975	0.003979
**STAT6**	signal transducer and activator of transcription 6, interleukin-4 induced	-1.83719	-0.877503	0.004125
**TICAM2**	toll-like receptor adaptor molecule 2	2.49223	1.31744	0.008159
**CEBPA**	CCAAT/enhancer binding protein (C/EBP), alpha	2.84794	1.50992	0.007799
**IRF4**	interferon regulatory factor 4	6.57144	2.71621	0.006589
**TLR6**	toll-like receptor 6	4.86554	2.2826	0.000368
**TLR8**	toll-like receptor 8	5.75342	2.52442	0.008690
**SPIB**	Spi-B transcription factor (Spi-1/PU.1 related)	9.2727	3.21299	0.005528
**LY9**	lymphocyte antigen 9	4.55617	2.18782	0.003384
**IRF1**	interferon regulatory factor 1	7.61361	2.92858	0.000708
**MAPK3**	mitogen-activated protein kinase 3	-3.07708	-1.62156	0.002192

**IFNGR1**	interferon gamma receptor 1	-2.04953	-1.03529	0.002680
**NKG7**	natural killer cell group 7 sequence	10.8226	3.43597	0.002100
**DDR1**	discoidin domain receptor tyrosine kinase 1	-61.8791	-5.95138	1.06E-07
**CD1D**	CD1d molecule	21.0715	4.39722	0.003838
**ITGA6**	integrin, alpha 6	-2.32014	-1.21421	0.007443
**IL32**	interleukin 32	6.78736	2.76285	0.000262
**LOC487977**		4.08612	2.03073	0.007017
**TRAC**		8.24489	3.0435	0.000971
**TRBC**		9.75154	3.28563	0.000360

Table S4 Differentially expressed genes between canine splenic HS and normal spleen

Name	Description	Fold Change	Log Fold Change	p-Value
**BCL2**	B-cell CLL/lymphoma 2	-1.77836	-0.83055	0.001292
**LIF**	leukemia inhibitory factor (cholinergic differentiation factor)	44.3333	5.47032	0.003848
**CD40LG**	CD40 ligand	-3.44829	-1.78588	0.005866
**COL1A1**	collagen, type I, alpha 1	50.7962	5.66665	0.000582
**CD80**	CD80 molecule	2.79475	1.48272	0.008924
**ITGA2B**	integrin, alpha 2b (platelet glycoprotein IIb of IIb/IIIa complex, antigen CD41)	-17.0	-4.08746	0.009997
**VEGFA**	vascular endothelial growth factor A	3.02273	1.59585	0.002840
**KIT**	v-kit Hardy-Zuckerman 4 feline sarcoma viral oncogene homolog	-9.62502	-3.26679	0.001240
**FN1**	fibronectin 1	11.265	3.49377	0.009275
**PDPN**	podoplanin	95.5835	6.57869	0.001683
**ADORA2A**	adenosine A2a receptor	-5.803	-2.5368	9.56E-05
**LGALS3**	lectin, galactoside-binding, soluble, 3	8.01632	3.00294	0.002599
**CAMP**	cathelicidin antimicrobial peptide	-28.7291	-4.84444	0.006506
**CLU**	clusterin	7.29521	2.86695	0.000326
**ELANE**	elastase, neutrophil expressed	-33.1289	-5.05002	0.006851
**CCL8**	chemokine (C-C motif) ligand 8	18.4285	4.20387	0.007504
**RPS6**	ribosomal protein S6	-2.86416	-1.51811	0.000778
**CDKN1A**	cyclin-dependent kinase inhibitor 1A (p21, Cip1)	3.22594	1.68972	0.008536
**IRF2**	interferon regulatory factor 2	-1.89327	-0.920877	0.007090
**TXNIP**	thioredoxin interacting protein	-4.96392	-2.31148	0.000439
**CD22**	CD22 molecule	-6.40709	-2.67967	0.000153
**IL17RB**	interleukin 17 receptor B	-14.6842	-3.87619	0.001064
**MME**	membrane metallo-endopeptidase	-12.4595	-3.63917	0.007275
**MAPK8**	mitogen-activated protein kinase 8	-2.06464	-1.04589	0.005829
**SPP1**	secreted phosphoprotein 1	204.397	7.67523	0.003494
**ALCAM**	activated leukocyte cell adhesion molecule	10.3747	3.375	0.007035
**TRAT1**	T cell receptor associated transmembrane adaptor 1	-18.2353	-4.18866	0.007663
**COL3A1**	collagen, type III, alpha 1	12.0788	3.5944	0.001892
**PIK3C2B**	phosphatidylinositol-4-phosphate 3-kinase, catalytic subunit type 2 beta	-3.85989	-1.94856	0.001425
**IDH2**	isocitrate dehydrogenase 2 (NADP+), mitochondrial	3.49579	1.80562	0.006049
**CYFIP2**	cytoplasmic FMR1 interacting protein 2	-6.5786	-2.71778	0.009522
**ICAM2**	intercellular adhesion molecule 2	-3.79182	-1.92289	0.000492
**CCL14**	chemokine (C-C motif) ligand 14	-11.3821	-3.5087	0.003906
**ERBB3**	v-erb-b2 avian erythroblastic leukemia viral oncogene homolog 3	-4.17808	-2.06284	0.006376
**TXK**	TXK tyrosine kinase	-13.3846	-3.7425	0.003369
**KDR**	kinase insert domain receptor (a type III receptor tyrosine kinase)	-3.02013	-1.59461	0.001701
**DUSP6**	dual specificity phosphatase 6	1.95343	0.966006	0.005138
**PIK3CG**	phosphatidylinositol-4,5-bisphosphate 3-kinase, catalytic subunit gamma	2.21151	1.14503	0.002000

**TOLLIP**	toll interacting protein	1.96169	0.972094	0.003392
**CTSW**	cathepsin W	-7.37253	-2.88216	0.009240
**GPR44**	G protein-coupled receptor 44	-5.11608	-2.35504	0.007083
**IGF2R**	insulin-like growth factor 2 receptor	3.25652	1.70333	0.001679
**CCR6**	chemokine (C-C motif) receptor 6	-2.18545	-1.12793	0.001861
**LOC484306**	leukocyte immunoglobulin-like receptor, subfamily B (with TM and ITIM domains), member 5-like	7.4436	2.896	0.002859
**POU2F2**	POU class 2 homeobox 2	-13.1667	-3.71882	0.005402
**AXL**	AXL receptor tyrosine kinase	-5.71019	-2.51354	0.008319
**FCER2**	Fc fragment of IgE, low affinity II, receptor for (CD23)	-8.25427	-3.04514	0.002226
**MS4A1**	membrane-spanning 4-domains, subfamily A, member 1	-19.3174	-4.27183	0.008726
**SPRY2**	sprouty homolog 2 (Drosophila)	-2.07024	-1.0498	0.000355
**ITGA5**	integrin, alpha 5 (fibronectin receptor, alpha polypeptide)	4.30278	2.10527	0.000786
**KRT8**	keratin 8	9.60603	3.26394	0.006619
**TOX**	thymocyte selection-associated high mobility group box	-5.39058	-2.43044	0.006583
**CD276**	CD276 molecule	3.35713	1.74723	0.008528
**RUNX1**	runt-related transcription factor 1	2.25885	1.17559	0.001495
**NPNT**	nephronectin	-4.58585	-2.19719	0.009780
**BTLA**	B and T lymphocyte associated	-8.88462	-3.15131	0.001500
**ITGAV**	integrin, alpha V	3.83767	1.94023	0.003356
**ITGA1**	integrin, alpha 1	-5.00288	-2.32276	0.007477
**CXCR5**	chemokine (C-X-C motif) receptor 5	-2.7619	-1.46566	0.009850
**IL12RB2**	interleukin 12 receptor, beta 2	-15.8333	-3.98489	0.005687
**CARD11**	caspase recruitment domain family, member 11	-4.45715	-2.15612	0.006619
**ITGAX**	integrin, alpha X (complement component 3 receptor 4 subunit)	11.5474	3.5295	0.002638
**IL4R**	interleukin 4 receptor	2.76571	1.46765	0.003661
**CCL7**	chemokine (C-C motif) ligand 7	25.5883	4.67741	0.007608
**IRAK1**	interleukin-1 receptor-associated kinase 1	5.04432	2.33466	0.002409
**CD14**	CD14 molecule	11.5148	3.52542	0.002404
**SHMT2**	serine hydroxymethyltransferase 2 (mitochondrial)	4.61804	2.20728	0.002446
**BCR**	breakpoint cluster region	-3.23187	-1.69237	4.52E-05
**CD19**	CD19 molecule	-12.0953	-3.59637	0.002684
**CTSG**	cathepsin G	-21.63	-4.43496	0.005245
**CD79B**	CD79b molecule, immunoglobulin-associated beta	-63.7211	-5.9937	0.004079
**CXCL14**	chemokine (C-X-C motif) ligand 14	3.05555	1.61143	0.007419
**LGALS1**	lectin, galactoside-binding, soluble, 1	2.43576	1.28437	0.001012
**TNFRSF12A**	tumor necrosis factor receptor superfamily, member 12A	8.85885	3.14712	0.004433
**PECAM1**	platelet/endothelial cell adhesion molecule 1	-3.82164	-1.93419	0.003200
**CD247**	CD247 molecule	-22.1304	-4.46796	0.002073
**PAX5**	paired box 5	-14.9643	-3.90345	0.006458
**FCRL2**	Fc receptor-like 2	-6.4737	-2.69459	0.008556

**TNFSF10**	tumor necrosis factor (ligand) superfamily, member 10	-3.87888	-1.95564	0.005736
**CDKN2A**	cyclin-dependent kinase inhibitor 2A (melanoma, p16, inhibits CDK4)	6.17582	2.62663	0.002168
**CEBPA**	CCAAT/enhancer binding protein (C/EBP), alpha	7.98526	2.99734	0.005970
**ICAM3**	intercellular adhesion molecule 3	-6.76744	-2.75861	0.004798
**SIGIRR**	single immunoglobulin and toll-interleukin 1 receptor (TIR) domain	1.78934	0.839429	0.001805
**ITGA6**	integrin, alpha 6	3.61905	1.85561	0.007668
**LOC102156836**	uncharacterized LOC102156836	-36.2684	-5.18064	0.001908
**LOC100856270**		2.58191	1.36844	0.007910

Table S5 Shared canine and human differentially expressed genes in HS

Name	Description	Mean Canine Log Fold Change	Mean Human Log Fold Change	Mean Canine Fold Change	Mean Human Fold Change
**BRCA1**	breast cancer 1, early onset	1.94365	2.075713	3.84678	4.215528
**BAX**	BCL2-associated X protein	1.22203	1.069803	2.33275	2.099146
**COL1A1**	collagen, type I, alpha 1	4.06228	4.485788	16.7058	22.40561
**KIT**	v-kit Hardy-Zuckerman 4 feline sarcoma viral oncogene homolog	-3.08746	-1.78783	-8.49998	-3.45295
**IGF1R**	insulin-like growth factor 1 receptor	-2.13276	-1.17824	-4.38556	-2.26301
**CLU**	clusterin	1.63158	-2.78725	3.09852	-6.90313
**CCL21**	chemokine (C-C motif) ligand 21	-3.2462	-5.58104	-9.48863	-47.8698
**TLR2**	toll-like receptor 2	1.69977	2.549572	3.24849	5.854604
**C2**	complement component 2	1.62824	1.332226	3.09136	2.51791
**TXNIP**	thioredoxin interacting protein	-2.56953	-1.79298	-5.93616	-3.46529
**KRT18**	keratin 18	-2.80649	-1.30549	-6.9958	-2.47167
**NRP1**	neuropilin 1	-1.51901	1.60223	-2.86594	3.036123
**SPP1**	secreted phosphoprotein 1	6.82605	5.12516	113.461	34.90011
**ICAM2**	intercellular adhesion molecule 2	-1.59873	-2.05973	-3.02877	-4.16907
**CCL14**	chemokine (C-C motif) ligand 14	-3.42488	-3.35504	-10.7397	-10.2322
**LTF**	lactotransferrin	-2.71359	-2.84419	-6.55952	-7.18104
**FCER2**	Fc fragment of IgE, low affinity II, receptor for (CD23)	-2.88402	-4.06862	-7.38204	-16.7794
**CPA3**	carboxypeptidase A3 (mast cell)	-2.75656	-2.81244	-6.75783	-7.02472
**C3AR1**	complement component 3a receptor 1	1.59831	1.755044	3.02788	3.375367
**NLRP3**	NLR family, pyrin domain containing 3	2.03932	2.221787	4.11052	4.66471
**CCL7**	chemokine (C-C motif) ligand 7	3.17408	8.854656	9.02596	462.9318
**CYBB**	cytochrome b-245, beta polypeptide (chronic granulomatous disease)	2.39805	1.817035	5.2709	3.523563
**CD14**	CD14 molecule	2.62111	2.346981	6.15223	5.087586
**CARD9**	caspase recruitment domain family, member 9	1.48938	2.435583	2.80768	5.409831
**TNFSF11**	tumor necrosis factor (ligand) superfamily, member 11	4.41844	-3.23665	21.3837	-9.42603
**CXCL14**	chemokine (C-X-C motif) ligand 14	2.15779	-3.4062	4.46231	-10.6015
**CLEC7A**	C-type lectin domain family 7, member A	2.81957	2.307241	7.05952	4.949356
**LY9**	lymphocyte antigen 9	1.31725	-2.19054	2.49191	-4.56475
**IGHM**		-3.71946	-3.13389	-13.1725	-8.77798
